# Efficacy of Anti-Interleukin-1 Therapeutics in the Treatment of Knee Osteoarthritis: A Systematic Review and Meta-Analysis of Randomized Controlled Trials from the Years 2000 to 2023

**DOI:** 10.3390/jcm13102859

**Published:** 2024-05-13

**Authors:** Michalina Knapik, Daniel Aleksander Żelazo, Karolina Osowiecka, Magdalena Krajewska-Włodarczyk

**Affiliations:** 1 Department of Rheumatology, School of Medicine, Collegium Medicum, University of Warmia and Mazury in Olsztyn, Wojska Polskiego 30, 10-719 Olsztyn, Poland; michalina24knapik@gmail.com (M.K.); zeldaniel95@gmail.com (D.A.Ż.); 2 Department of Psychology and Sociology of Health and Public Health, School of Public Health, University of Warmia and Mazury in Olsztyn, Warszawska 30, 10-082 Olsztyn, Poland; karolina.osowiecka@uwm.edu.pl

**Keywords:** anti-interleukin-1 therapeutics, meta-analysis, knee osteoarthritis

## Abstract

**Objectives**: This study aimed to evaluate the efficacy of anti-interleukin-1 therapeutics for treating knee osteoarthritis (KOA). Our research included interleukin-1 (IL-1) inhibitors, IL-1 antibodies and IL-1 receptor antagonists (IL-1 Ras). **Methods**: We systematically searched PubMed and Mendeley to find randomized control trials (RCTs) or clinical trials (CTs) of anti-interleukin-1 therapeutics in KOA from 2000 to 2023. The outcomes were changes in pain, function and stiffness scores. The research was conducted between November 2023 and January 2024. The risk of bias was assessed using Cochrane Risk of Bias tool RoB 2. **Results**: Analysis of the nine included studies showed a statistically significant difference in terms of the pain relief group (SMD = −0.20, 95% CI: −0.39 to −0.01, *p* = 0.0348), physical function improvement (SMD = −0.20, 95% CI: −0.39 to 0.00, *p* = 0.0479) and stiffness reduction (SMD = −0.22, 95% CI: −0.43 to 0.00, *p* = 0.0475) between anti-IL-1 therapeutics and placebo or nonsteroidal anti-inflammatory drugs (NSAIDs). However, when we separately analysed placebo and NSAIDs subgroups, the statistical significance was observed only in the placebo group. Our article was limited by the quality of the included RCTs. Two of the included trials were of poor methodological quality, and five showed selective reporting. **Conclusions**: The results of our study suggest that anti-IL-1 therapeutics might have better efficacy in KOA treatment than placebo or NSAIDs; yet, taking into account the limited availability of studies and data concerning anti-IL-1 in osteoarthritis treatment, we think that more high-quality RCTs on this subject are needed.

## 1. Introduction

Osteoarthritis (OA) is the most common chronic connective tissue inflammatory disease that might affect a single or several joints simultaneously [1,2,3]. Among the main clinical symptoms, the most significant are painful swelling, stiffness and progressive degeneration of the joints, leading to limited mobility and significant functional impairment. The most common localizations of the disease are knees and hips. There are over 654 million cases of knee OA (KOA) worldwide, and the prevalence is still increasing [1]. The number of cases of OA increases along with age and is more common in women, especially OA of the knees and small joints of the hands [4]. In recent years, an increase has also been observed in its cases among young people (below 40 years old) because of the increasing ratio of obesity in this group [5].

Currently, standard treatment approaches contain a wide range of oral medications embracing nonsteroidal anti-inflammatory drugs (NSAIDs) (as a first-line treatment), opioids, steroids and hyaluronic acid (HA) [6]. However, the prolonged use of nonsteroidal anti-inflammatory drugs or corticosteroids can lead to a series of side effects and limited therapeutic efficacy. 

The current treatments mainly concern pain control and reduction of inflammation, but no therapeutic strategy has been identified as a disease-modifying treatment. Therefore, identifying specific biomarkers that are useful in preventing, treating or distinguishing the stages of OA disease has become an immediate need in clinical practice. 

The molecular mechanisms of OA still remain incompletely understood. Presently, biological treatments such as platelet-rich plasma, bone marrow mesenchymal stem cells and autologous fragmented adipose tissue containing structural vascular fraction are commonly used. 

Due to the still disappointing effects of currently used forms of OA therapy and the recently discovered role of cytokines in the pathophysiology, more studies are being conducted about new therapeutic options for OA based on neutralization of IL-1 and TNF-alpha up-regulation of MMPs [7,8,9,10]. 

Taking into account the presently available subtypes of IL-1-targeted treatment, it is possible to distinguish between IL-1 inhibitors, IL-1 antibodies and IL-1 receptor antagonists (IL-1 Ras). 

Beginning the work on this meta-analysis, we found four meta-analyses with similar themes published in the years 2019–2023 [6,11,12,13]. However, the meta-analyses published so far have not included the most recent studies. Moreover, the newest one includes one retracted study [14]. In addition, none of these analyses examined the effect on stiffness, but only focused on pain and function. All four studies compared anti-IL-1 therapeutics only with placebo and not with NSAIDs. We decided to conduct this meta-analysis by including the newest studies as well as by examining the effect on stiffness and comparing anti-IL-1 therapeutics with NSAIDs. The previous meta-analyses also checked the safety of the examined drugs, while we concentrated only on efficacy.

Our study aims to analyse the newest available publications referring to biological treatment of OA, specifically with regard to anti-IL-1 therapeutics’ clinical effectiveness potential in the treatment of knee OA, evaluating their efficacy. Our hypothesis was that anti-IL-1 therapeutics would be more effective in the treatment of patients with KOA in terms of pain relief, physical function improvement and stiffness reduction compared to NSAIDs.

## 2. Methods

This study was conducted in accordance with PRISMA 2020 guidelines on issues related to the methodology of systematic reviews [15]. The study has not been registered at PROSPERO.

### 2.1. Search Strategy

Two independent reviewers conducted systematic literature research in PubMed and Mendeley. We used the following terms: “biological treatment of the knee osteoarthritis” OR “biological treatment of the osteoarthritis” OR “anti-interleukin-1 in the treatment of the knee osteoarthritis” OR “anti-interleukin-1 in the treatment of the osteoarthritis” OR “Diacerein in the treatment of the knee osteoarthritis” OR “Canakinumab in the treatment of the knee osteoarthritis” OR “Lutikizumab in the treatment of the knee osteoarthritis” OR “ABT-981 in the treatment of the knee osteoarthritis” OR “Orthokine in the treatment of the knee osteoarthritis” OR “Anakinra in the treatment of the knee osteoarthritis” OR “AMG-108 in the treatment of the knee osteoarthritis”.

### 2.2. Selection of Studies and Criteria

The qualification of the studies for our meta-analysis was based on the following criteria: 1. Randomized control trials (RCTs) or control trials (CTs). 2. Studies published between the years 2000 and 2023. 3. Patients diagnosed with KOA based on ACR criteria. 4. At least one of the subsequent issues: Western Ontario and McMaster Universities Osteoarthritis Index (WOMAC) pain score, WOMAC function score, WOMAC stiffness score, visual analogue scale (VAS) pain score.

We excluded studies that were neither RCTs nor CTs. Furthermore, trials not concerning knee joints as well as those which did not include any outcome indicators were also omitted.

Two independent reviewers conducted a search in PubMed and Mendeley, removed duplicates, reviewed the titles and abstracts and distinguished the excluded and included studies. Each reviewer checked the studies of the other and made a final decision. The research was conducted between November 2023 and January 2024.

### 2.3. Data Extraction

The data were extracted by two reviewers. We extracted characteristics of the publications, such as the first author, year of publication, country, company, medicine used in the treated group, types of intervention, time of follow-up in weeks, groups and subgroups. We also extracted characteristics of the patients, such as the number of participants in the treated and control groups, gender, age and body mass index (BMI). To compare the effectiveness of the treatment between studies, we extracted WOMAC pain, function and stiffness scores and VAS pain score before and after treatment in each study. Because the follow-up times were different in the analysed studies, we calculated the data from around a similar time frame. The most frequent data we extracted were from a 12-week follow-up. The shortest follow-up we took was 8 weeks and the longest was 52 weeks.

### 2.4. Risk of Bias Assessment

One reviewer used the Cochrane Risk of Bias tool RoB 2 to assess the risk of bias in the RCTs included in the meta-analysis. Each study was scored as having high, moderate (unclear) or low risk of bias in the following five domains: randomisation process, deviations from the intended interventions, missing outcome data, measurement of the outcome and selection of the reported result. 

### 2.5. Data Analysis and Statistical Methods

The mean and standard deviation differences in pain, stiffness and function were estimated for the intervention (IL) group and control group. There were calculated standardized mean differences (SMDs; Cohen’s d) with the corresponding 95% confidence intervals (CIs). The SMD was used in the meta-analysis to standardize the unit across studies, since there were different scales used in the included studies (the WOMAC and VAS to assess pain, the WOMAC scale for stiffness and function evaluation). SMDs smaller than zero indicated a beneficial effect for IL groups. SMDs of 0.2, 0.5 and 0.8 were considered small, medium and large, respectively [16]. Heterogeneity was measured by Cochran’s Q. The T^2^ and I^2^ statistics describe, respectively, the variation and percentage of variation across studies. The random effects model was used due to the heterogeneity of the analysed studies. The overall effect size was shown in forest plots. The analyses in subgroups were performed to understand the effect of different types of control group and the method of anti-IL-1 administration on pain, function and stiffness. Meta-regression was used to identify independent significant predictors of heterogeneity between studies.

A *p*-value of <0.05 was considered to be significant. The data analysis was conducted using Statistica (data analysis software), version 13. http://statistica.io, TIBCO Software Inc., Krakow, Poland, 2017 (accessed on 1 January 2024).

## 3. Results

### 3.1. Literature Search

**Figure 1:**
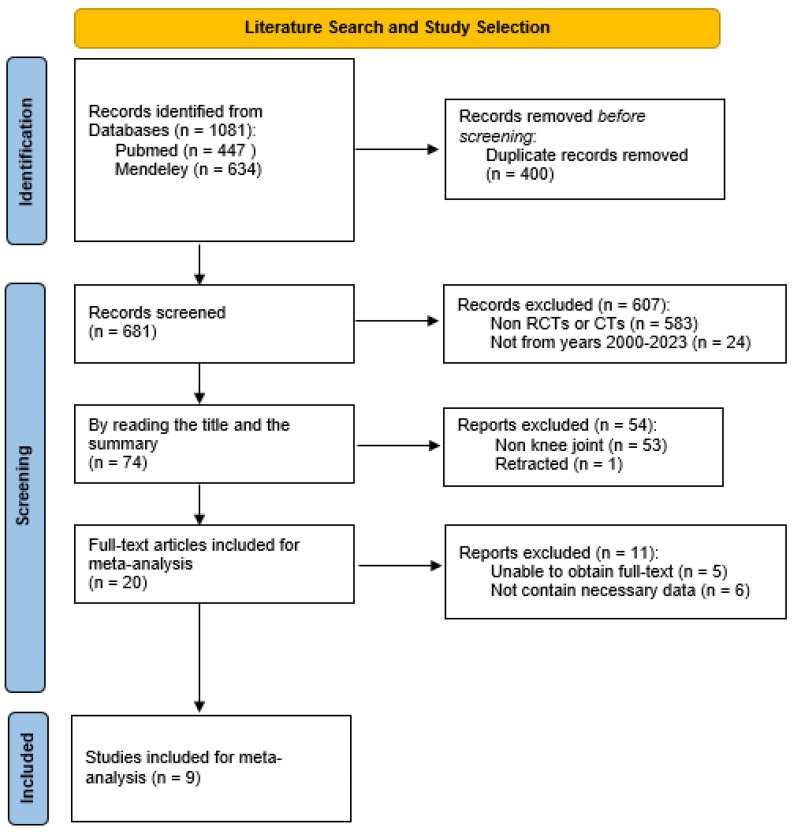
Flow chart of the literature search and study selection.

Two reviewers found a total of 1081 potential studies matching the terms used for the research. After the elimination of duplicates and all the publications which did not fit our subject, then limiting ourselves to RCTs and CTs only and to publications from the years 2000 to 2023, we finally extracted nine RCTs [17,18,19,20,21,22,23,24,25] which we included in our meta-analysis. The process of the study selection is presented in Figure 1.

### 3.2. Study Characteristics

The detailed characteristics of the included studies and the patients included in each study are presented in Table 1 and Table 2. All of the included studies are RCTs published between the years 2000 and 2023. The anti-IL-1 therapeutics included in these studies could be classified into three categories: IL-1 inhibitors, IL-1 Ras and IL-1 antibodies. Placebo was used as a control group in six studies [20,21,22,23,24,25], of which one was physiological saline [23] and the others had not explained what was used as a placebo. In the other three studies [17,18,19], they used NSAIDs as a control group, out of which one was piroxicam [18], and in two other studies celecoxib was used [17,19]. The total sample size from all the studies included was 1252 patients in the treatment group (anti-IL-1 therapeutics) and 778 patients in the control group.

### 3.3. Risk of Bias

**Figure 2:**
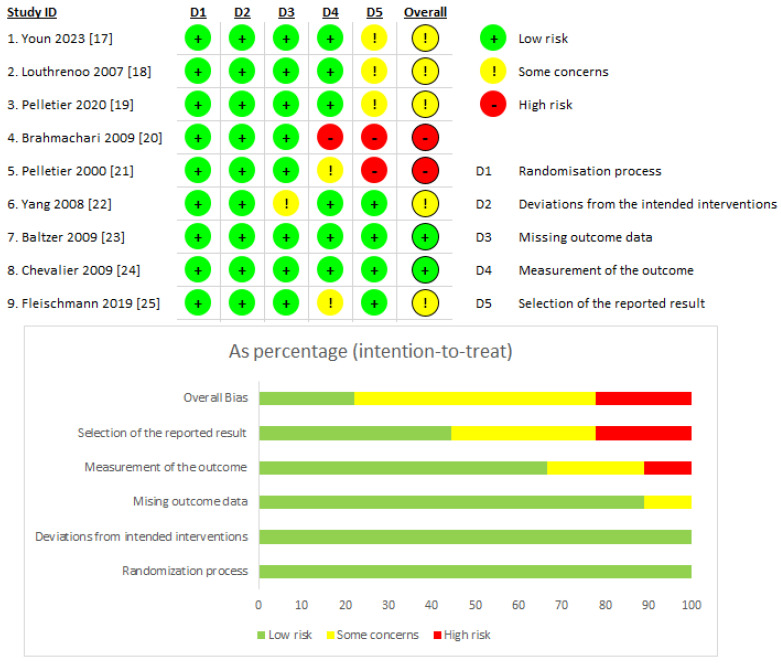
Risk of bias assessment results [17,18,19,20,21,22,23,24,25].

The outcomes of the risk of bias assessment are presented in Figure 2. Out of the nine studies, two studies turned out to have a high risk of bias [20,21], five studies were judged to have a moderate risk of bias [17,18,19,21,25], and two studies were found to have a low risk of bias [23,24]. In all of the studies [17,18,19,20,21,22,23,24,25], there was an appropriate randomisation process and there were no deviations from the intended interventions. In one of the studies [22], some concerns arose about missing outcome data. Another concern was about the measurement of the outcome in two studies [21,25], as well as the selection of the reported result in three studies [17,18,19]. A high risk of bias was marked in one study [20] for the measurement of the outcome and in two studies [20,21] for the selection of the reported result.

### 3.4. Knee Pain Scores

**Figure 3:**
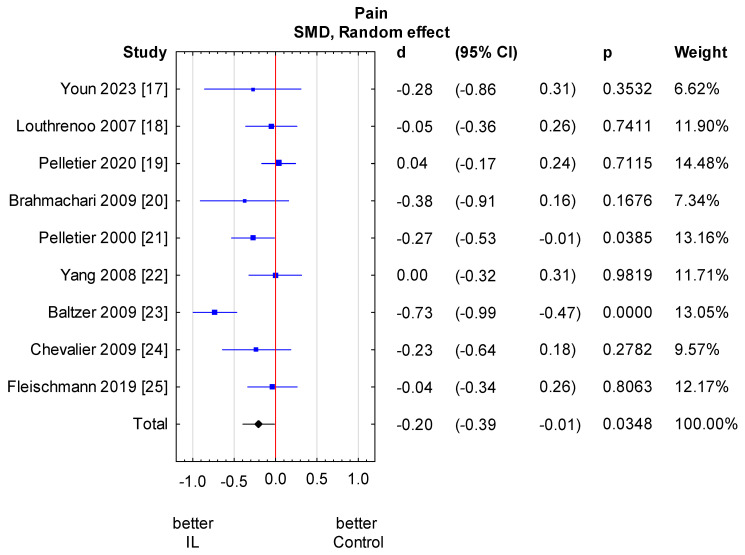
Knee pain score results. Heterogeneity: Q = 25.3, df = 8 (*p* = 0.001); T2 = 0.05, I2 = 68.4%. CI—confidence interval [17,18,19,20,21,22,23,24,25].

**Figure 4:**
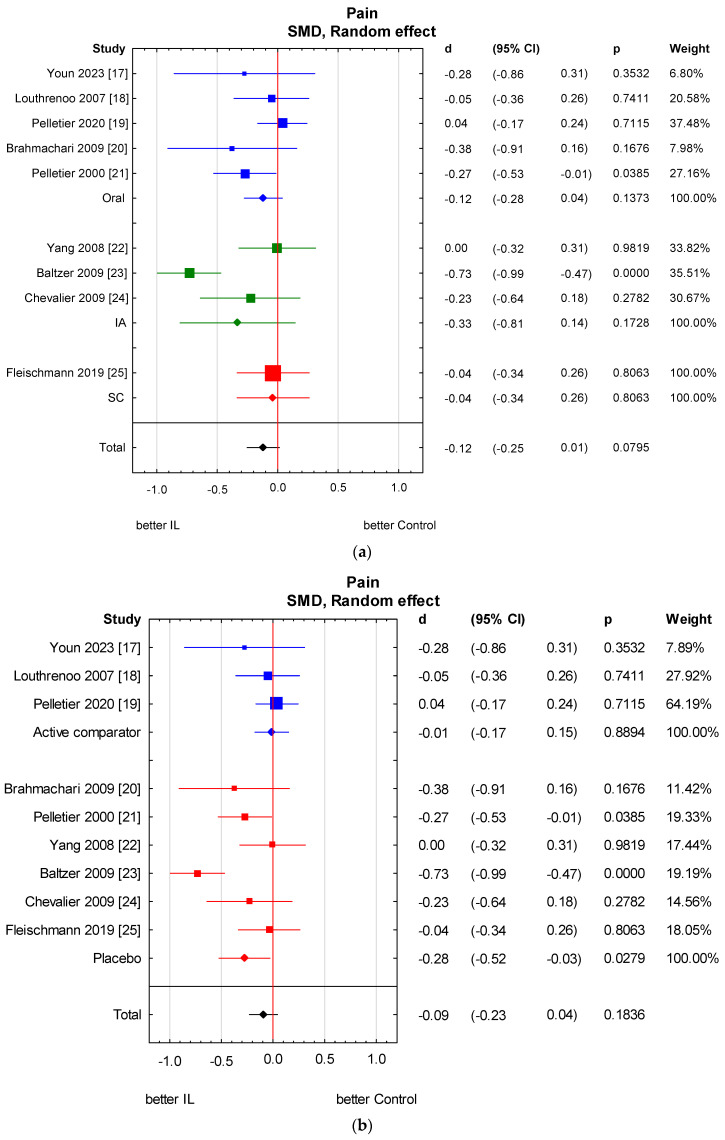
Knee pain score results in subgroups: (**a**) method of administration; (**b**) type of control group. (**a**) Heterogeneity: Q Oral = 3.7, df = 4 (*p* = 0.45); Q IA = 1.6, df = 2 (*p* = 0.44); Q SC not applicable; Q Total = 1.05, df = 2 (*p* = 0.59). (**b**) Heterogeneity: Q Placebo = 4.1, df = 5 (*p* = 0.54); Q Active comparator = 1.1, df = 2 (*p* = 0.58); Q Total = 3.1, df = 1 (*p* = 0.08). CI—confidence interval [17,18,19,20,21,22,23,24,25].

Among the nine studies, six studies [17,18,21,23,24,25] assessed pain scores with the WOMAC and three studies [19,20,22] with the VAS. We found a statistically significant pain reduction in the anti-IL-1 therapeutic group compared to the control group (SMD = −0.20, 95% CI: −0.39 to −0.01, *p* = 0.0348). However, it oscillates around the significance threshold. There is relevant heterogeneity across studies, but we were not able to explain it with the analysed variables. The analysis was performed in subgroups taking into consideration the methods of administering anti-IL-1 therapeutics and types of control groups. There is no considerable heterogeneity in the analysed subgroups as well as between subgroups. Variables: method of administration and type of control group do not significantly impact the heterogeneity of the studies. We also made a meta-regression. The detailed analysis is shown in Figure 3 and Figure 4 and Table 3 and Table 4.

### 3.5. Knee Function Scores

**Figure 5:**
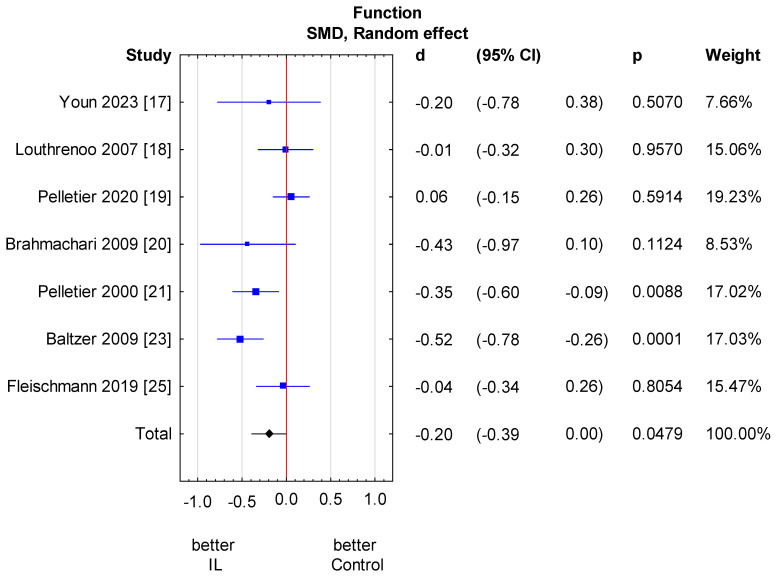
Knee function score results. Heterogeneity: Q = 16.2, df = 6 (*p* = 0.01); T2 = 0.04, I2 = 63.0%. CI—confidence interval [17,18,19,20,21,23,25].

**Figure 6:**
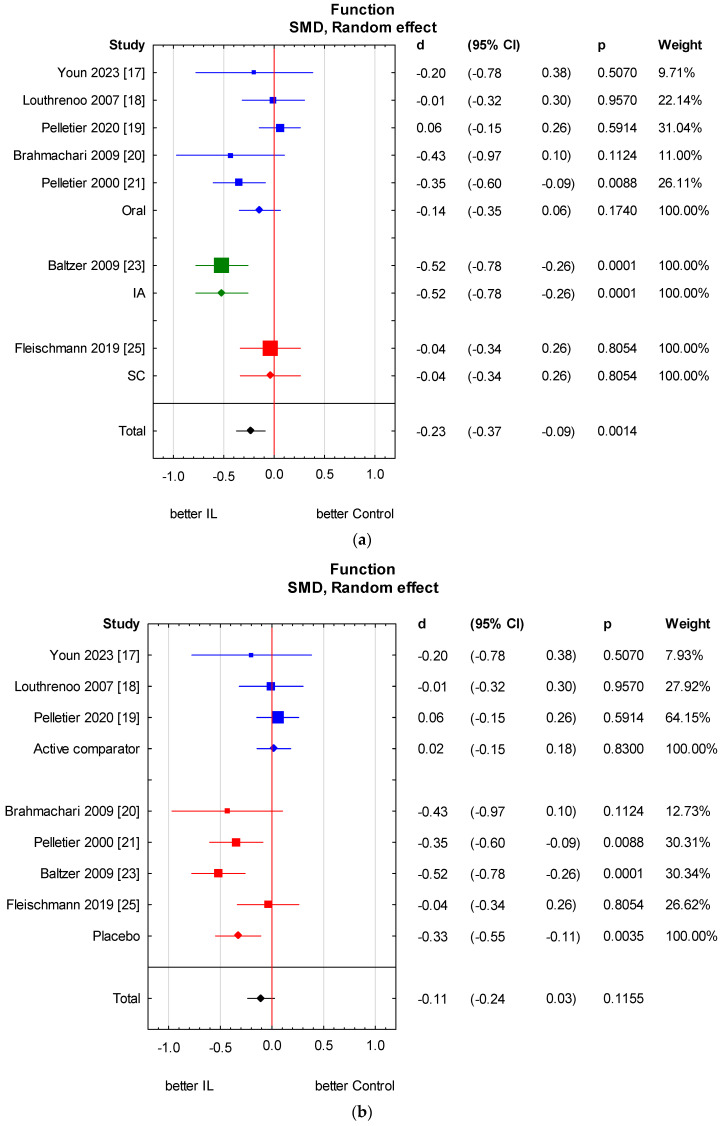
Knee function score results in subgroups: (**a**) method of administration; (**b**) type of control group. (**a**) Heterogeneity: Q Oral = 3.4, df = 4 (*p* = 0.50); Q IA not applicable; Q SC not applicable; Q Total = 7.1, df = 2 (*p* = 0.03). (**b**) Heterogeneity: Q Placebo = 2.8, df = 3 (*p* = 0.42); Q Active comparator = 0.7, df = 2 (*p* = 0.71); Q Total = 6.1, df = 1 (*p* = 0.01). CI—confidence interval [17,18,19,20,21,23,25].

Only seven out of nine studies assessed physical function scores [17,18,19,20,21,23,25]. All of them used the WOMAC function score. We found a statistically significant upswing of physical function in the anti-IL-1 therapeutic group compared to the control group (SMD = −0.20, 95% CI: −0.39 to 0.00, *p* = 0.0479). There is relevant heterogeneity across studies. We performed the analysis in subgroups taking into account the methods of administration of anti-IL-1 therapeutics and types of control groups. There is no considerable heterogeneity in the analysed subgroups; however, it remains relevant between subgroups. A significant independent predictor of variability is the control group type. In the placebo group, a considerably beneficial effect related to function is observed. We also made a meta-regression. The detailed analysis is shown in Figure 5 and Figure 6 and Table 5 and Table 6.

### 3.6. Knee Stiffness Scores

**Figure 7:**
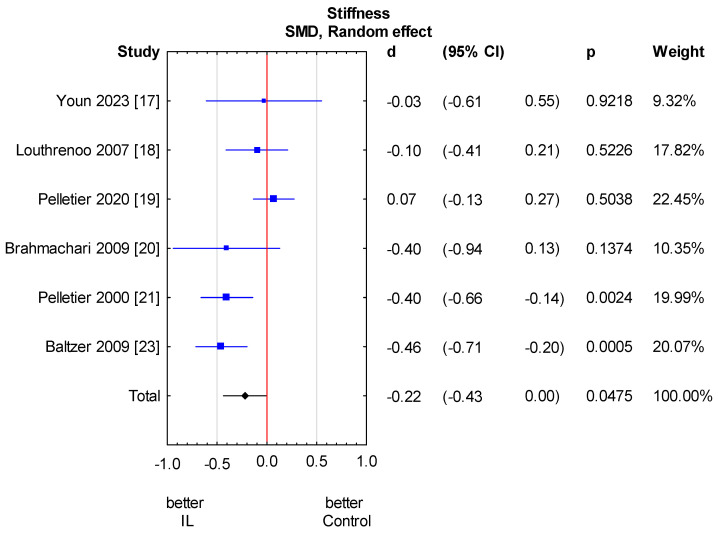
Knee stiffness score results. Heterogeneity: Q = 14.1, df = 5 (*p* = 0.01); T2 = 0.04, I2 = 64.6%. CI—confidence interval [17,18,19,20,21,23].

**Figure 8:**
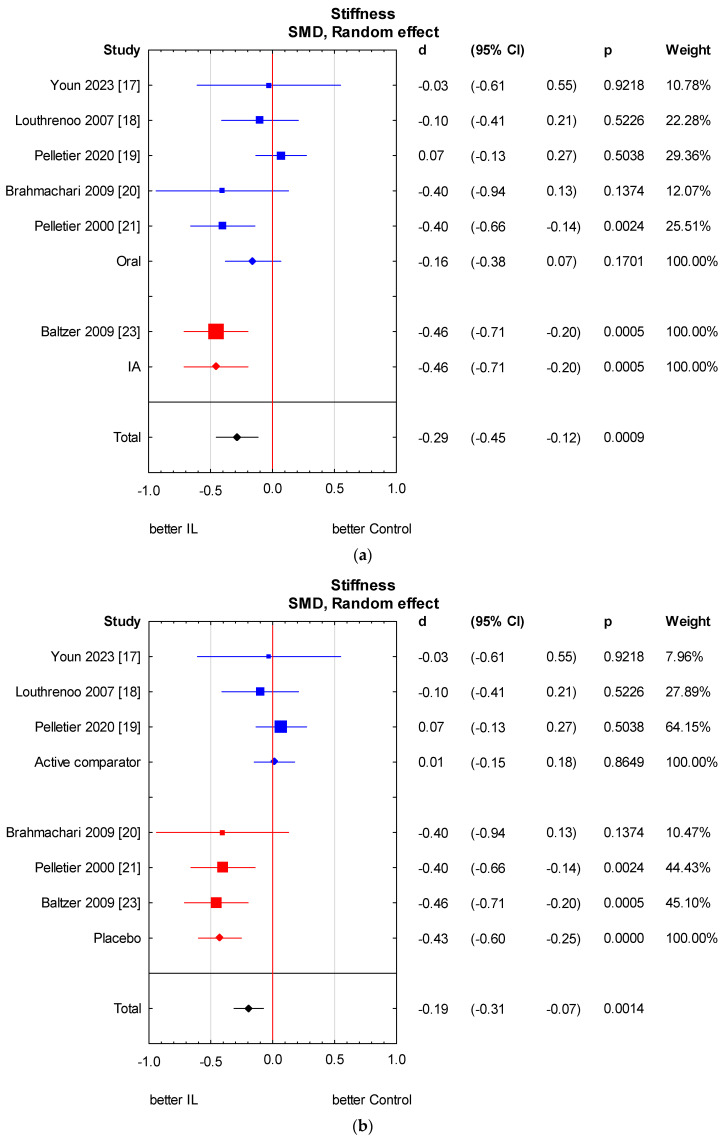
Knee stiffness score results in subgroup: (**a**) method of administration; (**b**) type of control group. (**a**) Heterogeneity: Q Oral = 3.1, df = 4 (*p* = 0.54); Q IA not applicable; Q Total = 3.0, df = 1 (*p* = 0.09). (**b**) Heterogeneity: Q Placebo = 0.1, df = 2 (*p* = 0.95); Q Active comparator = 0.8, df = 2 (*p* = 0.66); Q Total = 13.2, df = 1 (*p* < 0.001). CI—confidence interval [17,18,19,20,21,23].

Among the nine studies, only six assessed stiffness scores [17,18,19,20,21,23]. All of them used the WOMAC stiffness score. We found a statistically significant stiffness reduction in the anti-IL-1 therapeutic group compared to the control group (SMD = −0.22, 95% CI: −0.43 to 0.00, *p* = 0.0475). However, it oscillates around the significance threshold. There is relevant heterogeneity across studies. We performed the analysis in subgroups taking into account the methods of administration of anti-IL-1 therapeutics and types of control groups. There is no considerable heterogeneity in the analysed subgroups; however, it remains relevant between subgroups. A significant independent predictor of variability is the control group type. In the placebo group, a considerably beneficial effect related to stiffness is observed. We also made a meta-regression. The detailed analysis is shown in Figure 7 and Figure 8 and Table 7 and Table 8.

## 4. Discussion

This meta-analysis comprehensively evaluated the efficacy of three main categories of proinflammatory interleukin-1 direct therapeutics, including IL-1 receptor antagonists, anti-IL-1 antibodies and IL-1 inhibitors, in patients with knee osteoarthritis. 

The most frequently observed lesions are the consequences of mechanical overstraining of the joints, which leads to disturbance of the biomechanics of the joint and progressive degeneration of the cartilage extracellular matrix (ECM), related to the change between synthesis and degeneration of its components [3,26]. Mechanical stimulation of chondrocytes provokes the secretion of cytokines such as interleukin-1 (IL-1) and (tumour necrosis factor-α) TNF-α, which stimulate the secretion of proteolytic enzymes such as metalloproteinases (MMPs) which destroy the tissues of the joint. Cytokines also weaken the chondrocyte compensatory synthesis pathway [27]. At the beginning of the disease, active reconstructive mechanisms occur—intensified production of collagen type II and proteoglycans, which leads to cartilage reinforcement [28,29]. Moreover, IGF-1 and TGF-β also play a major role in reparative mechanisms [11,30,31,32,33]. When the cartilage runs out of its reparative abilities, it starts to change its biochemical structure—loses water and proteoglycans, which makes it more vulnerable to injury. Destruction of the cartilage leads to changes in the structure of the joint—narrowing of the joint space, sclerotization, osteophytes and hyperplasia of the synovial membrane [34,35]. 

This review highlights the role of IL-1, which seems to be strictly associated with three main pathways involved in OA development: chondrocyte lifespan and apoptosis, cartilage ECM components synthesis and inflammatory processes [3]. The ECM’s composition is constantly remodelled by the action of chondrocytes under the effect of MMPs triggered by pro-inflammatory factors, such as IL-1β and TNF-α, followed by the degradation of its main constituents such as COL2A1 and aggrecan [3,36]. 

There is also an increasing number of reports highlighting the role of IL-1β as a key cytokine in angiogenesis [36].

Noteworthy is the fact that IL-1 subtypes have considerably expanded in recent years [36,37]. The IL-1R family has also expanded to nine specific genes including coreceptors, binding proteins, inhibitory receptors and other target points on the IL-1-related signal transduction pathway [31,38,39]. Thus, the IL-1 pathway remains a promising target for OA pharmacological therapy.

IL-1 antibodies are therapeutic human dual-variable-domain immunoglobulins having the ability to neutralize human IL-1α and/or IL-1β subtypes [12]. Interleukin-1 receptor antagonists (IL-1Ras) are members of the IL-1 family binding to IL-1 receptors, which is a crucial anti-inflammatory protein in OA pathogenesis. IL-1 inhibitors are defined as molecules that inhibit the synthesis and activity of IL-1.

Since all of the assimilated studies were double-blinded randomized placebo-controlled trials, our subgroup analyses associated with the mechanism of action allowed us to make an indirect comparison for these three main anti-IL-1 therapeutic categories.

To update the therapeutic evidence of anti-IL-1 medications in KOA treatment, nine RCTs were included, involving three main anti-IL-1 subgroups based on the mechanism of their action: lutikizumab (ABT981) was reported in one study as IL-1 monoclonal antibody [25], Orthokine as well as Anakinra were described in three studies as IL-1 -Ras [22,23,24], whereas reports of Diacerein were published in five articles as an IL-1 inhibitor [17,18,19,20,21].

The aggregated results indicate a significant difference compared to the control group in terms of relieving pain, stiffness and functional improvement. However, the analysis in subgroups shows the relevant difference only when anti-IL-1 drugs are compared to placebo but not when compared to NSAIDs. That indicated that anti-IL-1 therapeutics may have a comparable level of effectiveness in KOA treatment to NSAIDs. This represents a promising perspective for a group of patients struggling with KOA and accustomed to a standard treatment strategy of taking large amount of NSAIDs to reduce pain. As is well known, chronic intake of NSAIDs is associated with serious side effects such as gastric ulcers, liver damage or analgesic nephropathy. Taken in high doses over several days they can even induce acute kidney injury. Taking this into account, anti-IL-1 therapeutics may be an opportunity for better OA symptoms control, and at the same it may help with side-effects prevention. Obviously, more studies are needed on this group of drugs, and it is necessary to check the safety profile of anti-IL-1 medications compared to NSAIDs, because, as with all drugs, anti-IL-1 therapeutics are also capable of causing adverse effects. From the patient’s point of view, noteworthy is the fact that IL-1 inhibitors significantly exceed the cost of NSAIDs treatment. 

Our results are parallel to those from the meta-analysis made by Lizhi Yu et al. in February 2023 [6]. However, there are two-fold main differences between our meta-analyses. First of all, we implemented the stiffness evaluation into our study as an additional factor. Secondly, we focused only on the efficacy of anti-IL-1 therapeutics, while Lizhi Yu et al. also took adverse events into account. Comparing to the meta-analysis from 2023 we excluded three RCTs which included only data concerning safety and not efficacy [40,41,42]. Moreover, one of the publications they included was retracted in June 2023 [14]. 

Nevertheless, our results are not in line with the current American College of Rheumatology (ACR) and European Alliance of Associations for Rheumatology (EULAR) guidelines for osteoarthritis treatment [43,44].

Our research has several strengths. The study selection and data extraction, including quality appraisal, were performed independently by two authors and were discussed with a third author specializing in rheumatology, thus minimizing bias and the occurrence of transcriptional errors. A comprehensive methodological quality evaluation of all the included studies was performed. 

Currently, new studies about the use of anti-IL-1 therapeutics in the treatment of osteoarthritis are still emerging, as the topic still needs to be explored. A very recent study is the Australian DICKENS study by Cai et al. [45]. This is a multicentre, randomized, double-blinded, placebo-controlled trial about Diacerein for knee osteoarthritis with effusion-synovitis. The study includes 262 patients and assesses the change in pain, function, stiffness and effusion-synovitis volume after 24 weeks of taking Diacerein compared to placebo. Diacerein was administered orally, at a dose of 50 mg twice daily for the first two weeks and then 100 mg twice daily until the end of the study. Variables were assessed using the VAS and WOMAC scales and MRI examination. The study also assessed adverse events of Diacerein compared to placebo. They concluded that compared to placebo, Diacerein did not significantly improve knee pain or effusion-synovitis and that adverse events were more frequent in the Diacerein group, so they do not support the use of Diacerein in treating KOA patients with effusion-synovitis. We did not use the study in our meta-analysis as it is a very recent study and only an abstract of the study is currently available which does not contain enough data. However, we believe it is worthy of consideration, since it is the largest trial to date, and should be included in the next such meta-analysis.

## 5. Limitations

This meta-analysis has a couple of limitations. Like most systematic assessments, our article was limited by the quality of the included RCTs. Two of the included trials were of poor methodological quality [20,21], and five showed selective reporting [17,18,19,22,25]. 

The amount of available research studies on anti-IL-1 therapeutics in KOA treatment is not sufficient to come to an agreement on the efficacy of ILs for KOA, which indicates that more randomized controlled trials and meta-analyses are necessary to update the anti-IL-1 therapeutic evidence in the treatment of KOA.

The studies included were heterogeneous in terms of the dosages and method of administration. IL-1Ras (Orthokine and Anakinra) was administered intraarticularly, IL-1 inhibitor (Diacereine) orally and novel IL-1α/β Ab (ABT981/Lutikizumab) subcutaneously. These factors may constitute certain limitations for clinical appraisal and account for the different clinical outcomes in patients. The follow-up time among the included studies also remained variable, ranging from 8 to 52 weeks. 

In our meta-analysis, all of the included studies report knee pain outcomes, whilst six out of nine display knee stiffness scores, and seven trials highlight knee function scores. All the contributing factors described above make it impossible to draw conclusions about the effect of anti-IL-1 therapeutics compared to control treatment for KOA. 

## 6. Conclusions

The results of our study suggest that anti-IL-1 therapeutics might have better efficacy in KOA treatment than placebo or NSAIDs. In all three parameters (pain, physical function and stiffness) that we checked, change was statistically significant. 

However, our results are limited by the quality of the included RCTs. 

In conclusion, due to the differences between the results of our meta-analysis and the ACR and EULAR guidelines, and taking into account the limited availability of studies and data about anti-IL-1 in osteoarthritis treatment, more high-quality RCTs on this subject are needed to verify if this is a proper method of KOA treatment and if it can be added into standardized guidelines.

## Figures and Tables

**Table 1:** Characteristics of the studies.

No.	Study and Year	Type	Country	Company	Medicine	Intervention	Follow-Up, Weeks	Group	Subgroups	Sample Size, n	
										Treat	Control
1	Youn 2023 [17]	RCT	South Korea	NA	Diacerein (IL-1 inhibitor)	Oral, 50 mg twice a day for 12 weeks	16	50 mg	1	26	27
2	Louthrenoo 2007 [18]	RCT	Thailand	TRB Chemedica International SA	Diacerein (IL-1 inhibitor)	Oral, 50 mg once a day for 16 weeks	24	50 mg	1	82	79
3	Pelletier 2020 [19]	Muliticentre RCT	Canada	NA	Diacerein (IL-1 inhibitor)	Oral, 50 mg once a day for 1 month and twice daily thereafter	26	50 mg	1	183	187
4	Brahmachari 2009 [20]	RCT	India	NA	Diacerein (IL-1 inhibitor)	Oral, once a day for the first 10 days, 50 mg after meals, lasting for 8 weeks	12	50 mg	1	28	27
5	Pelletier 2000 [21]	Muliticentre RCT	France	Les Laboratoires Negma	Diacerein (IL-1 inhibitor)	Oral, twice a day for 16 weeks	12	50 mg	1	126	124
								100 mg	2	110	
								150 mg	3	120	
6	Yang 2008 [22]	RCT	Netherlands	NA	Orthokine (IL-1 Ras)	IA on days 0,3,7,10,14 and 21	48	NA	1	80	73
7	Baltzer 2009 [23]	Muliticentre RCT	Germany	NA	Orthokine (IL-1 Ras)	IA, twice a week, lasting for 3 weeks	26	NA	1	134	107
8	Chevalier 2009 [24]	Muliticentre RCT	France	Amgen Inc	Anakinra (IL-1 Ras)	Single IA	12	50 mg	1	34	69
								150 mg	2	67	
9	Fleischmann 2019 [25]	RCT	USA	AbbVie Inc	Lutikizumab (IL-1 antibody)	SC, once every 2 weeks, lasting for 50 weeks	52	25 mg	1	89	85
								100 mg	2	85	
								200 mg	3	88	

NA—not available.

**Table 2:** Characteristics of the included patients.

			Female, n (%)		Age (Years), Mean ± SD		BMI (kg/m^2^), Mean ± SD	
No.	Study and Year	Subgroup	Treat	Control	Treat	Control	Treat	Control
1	Youn 2023 [17]	1	22 (84.62)	22 (81.48)	64.92 ± 7.85	65.85 ± 7.85	24.6 ± 2.75	25.75 ± 3.3
2	Louthrenoo 2007 [18]	1	73 (89)	73 (92.4)	54 ± 6.2	54 ± 7	27.4 ± 3.4	26.3 ± 3.6
3	Pelletier 2020 [19]	1	133 (72.7)	141 (75.4)	63.7 ± 6.3	64.4 ± 7.0	31.2 ± 5.5	30.0 ± 5.0
4	Brahmachari 2009 [20]	1	26 (92.8)	20 (74)	45.5 ± 10.52	53 ± 11.85	25.3 ± 3.63	20 ± 3.7
5	Pelletier 2000 [21]	1	105 (83.3)	98 (79)	62.95 ± 8.41	64.5 ± 8.65	31.63 ± 5.5	31.05 ± 5.35
		2	83 (75.5)		64.22 ± 8.02		31.73 ± 6.21	
		3	96 (80)		62.27 ± 10.18		30.99 ± 5.88	
6	Yang 2008 [22]	1	31 (39)	30 (41)	54 ± 11	53 ± 11	27 ± 5	28 ± 14
7	Baltzer 2009 [23]	1	65 (48.5)	68 (63.6)	53.8 ± 12.2	60.3 ± 10.7	NA	NA
8	Chevalier 2009 [24]	1	17 (50)	44 (64)	63.3 ± 9.8	62.2 ± 10	NA	NA
		2	46 (69)		62.6 ± 9.4		NA	
9	Fleischmann 2019 [25]	1	63 (70.8)	52 (61.2)	61.6 ± 7.5	59.5 ± 8.9	28.7 ± 3.8	28.6 ± 3.6
		2	53 (62.4)		60.2 ± 8.2		29 ± 3.5	
		3	57 (64.8)		59.1 ± 10.3		28.7 ± 3.5	

NA—not available. SD—standard deviation.

**Table 3:** Knee pain score results. SD—standard deviation.

		IL			Control		
No.	Study and Year	N	Mean Difference	SD Difference	N	Mean Difference	SD Difference
1	Youn 2023 [17]	24	−4.21	3.23	22	−3.18	4.23
2	Louthrenoo 2007 [18]	82	36.40	22.61	79	37.50	19.44
3	Pelletier 2020 [19]	183	−2.40	2.60	187	−2.50	2.60
4	Brahmachari 2009 [20]	28	−24.50	39.08	27	−10.00	38.13
5	Pelletier 2000 [21]	110	−58.80	92.50	124	−33.90	90.50
6	Yang 2008 [22]	80	−16.05	33.32	73	−15.93	32.15
7	Baltzer 2009 [23]	134	−2.85	2.28	107	−1.25	2.06
8	Chevalier 2009 [24]	34	−27.3	29.9	69	−20.7	28.5
9	Fleischmann 2019 [25]	88	−12.2	60.69	85	−10	57.19

**Table 4:** Meta-regression model for pain (Cohen’s d; random effect). CI—confidence interval.

Independent Variables	b	Standard Error	95% CI	Z	*p*
Method of administration					
Oral	−0.08	0.18	−0.43–0.27	−0.44	0.66
IA	−0.11	0.16	−0.43–0.20	−0.71	0.48
SC	0.19	0.21	−0.21–0.60	0.94	0.35
Type of control group					
Active comparator	0.13	0.14	−0.14–0.39	0.93	0.35
Placebo	−0.13	0.14	−0.39–0.14	−0.93	0.35

R^2^ = 0.34%, I^2^ = 68.4%.

**Table 5:** Knee function score results. SD—standard deviation.

		IL			Control		
No.	Study and Year	N	Mean Difference	SD Difference	N	Mean Difference	SD Difference
1	Youn 2023 [17]	24	−10.75	11.28	22	−8.14	15.20
2	Louthrenoo 2007 [18]	82	31.60	24.69	79	31.80	22.27
3	Pelletier 2020 [19]	183	−27.10	39.00	187	−29.30	39.80
4	Brahmachari 2009 [20]	28	−267.70	384.63	27	−96.00	408.15
5	Pelletier 2000 [21]	110	−193.30	318.00	124	−85.80	304.40
7	Baltzer 2009 [23]	134	−2.81	3.18	107	−1.17	3.14
9	Fleischmann 2019 [25]	88	−39.70	197.87	85	−32.90	162.72

**Table 6:** Meta-regression model for Function (Cohen’s d; random effect). CI—confidence interval.

Independent Variables	b	Standard Error	95% CI	Z	*p*
Method of administration					
Oral	−0.06	0.10	−0.26–0.15	−0.54	0.59
IA	−0.21	0.11	−0.43–0.00	−1.95	0.05
SC	0.27	0.12	0.04–0.50	2.29	0.02
Type of control group					
Active comparator	0.19	0.07	0.05–0.33	2.62	0.009
Placebo	−0.19	0.07	−0.33–(−0.05)	−2.62	0.009

R^2^ = 100%, I^2^ = 63.0%.

**Table 7:** Knee stiffness score results. SD—standard deviation.

		IL			Control		
No.	Study and Year	N	Mean Difference	SD Difference	N	Mean Difference	SD Difference
1	Youn 2023 [17]	24	−1.00	1.44	22	−0.95	1.99
2	Louthrenoo 2007 [18]	82	35.40	28.85	79	38.10	24.43
3	Pelletier 2020 [19]	183	−3.60	5.00	187	−4.00	6.40
4	Brahmachari 2009 [20]	28	−49.00	79.40	27	−11.00	106.84
5	Pelletier 2000 [21]	110	−27.30	42.30	124	−10.30	42.40
7	Baltzer 2009 [23]	134	−2.79	3.57	107	−1.09	3.92

**Table 8:** Meta-regression model for Stiffness (Cohen’s d; random effect). CI—confidence interval.

Independent Variables	b	Standard Error	95% CI	Z	*p*
Method of administration					
Oral	0.03	0.09	−0.15–0.20	0.30	0.76
IA	−0.03	0.09	−0.20–0.15	−0.30	0.76
Type of control group					
Active comparator	0.21	0.07	0.07–0.35	2.87	0.004
Placebo	−0.21	0.07	−0.35–(−0.07)	−2.87	0.004

R^2^ = 100%, I^2^ = 64.6%.
